# Multi-Scale Contrastive Learning with Hierarchical Knowledge Synergy for Visible-Infrared Person Re-Identification

**DOI:** 10.3390/s25010192

**Published:** 2025-01-01

**Authors:** Yongheng Qian, Su-Kit Tang

**Affiliations:** 1 Faculty of Applied Sciences, Macao Polytechnic University, Macao SAR 999078, China; yongheng.qian@mpu.edu.mo; 2 Department of Mechatronics and Information Engineering, Zunyi Vocational and Technical College, Zunyi 563000, China

**Keywords:** cross-modality, deep supervision, contrastive learning, knowledge synergy, person re-identification

## Abstract

Visible-infrared person re-identification (VI-ReID) is a challenging cross-modality retrieval task to match a person across different spectral camera views. Most existing works focus on learning shared feature representations from the final embedding space of advanced networks to alleviate modality differences between visible and infrared images. However, exclusively relying on high-level semantic information from the network’s final layers can restrict shared feature representations and overlook the benefits of low-level details. Different from these methods, we propose a multi-scale contrastive learning network (MCLNet) with hierarchical knowledge synergy for VI-ReID. MCLNet is a novel two-stream contrastive deep supervision framework designed to train low-level details and high-level semantic representations simultaneously. MCLNet utilizes supervised contrastive learning (SCL) at each intermediate layer to strengthen visual representations and enhance cross-modality feature learning. Furthermore, a hierarchical knowledge synergy (HKS) strategy for pairwise knowledge matching promotes explicit information interaction across multi-scale features and improves information consistency. Extensive experiments on three benchmarks demonstrate the effectiveness of MCLNet.

## 1. Introduction

Person re-identification (ReID) is a key technology in intelligent surveillance, social security, and national governance, designed to identify persons with the same identity across non-overlapping camera views captured at different times [1,2]. Currently, most research [3,4,5,6,7,8] focuses on visible-visible person re-identification (VV-ReID). VV-ReID is a single-modality retrieval task, matching images captured exclusively by visible cameras. However, visible cameras often fail to capture effective appearance information under low-light conditions or at night, limiting the broader application of VV-ReID.

With the rapid advancement of smart imaging sensors, cameras that automatically switch between visible and infrared modes based on lighting conditions have become widely deployed. Infrared (IR) images captured in infrared mode can be matched with visible (RGB) images. Consequently, ref. [9] defined the visible-infrared person re-identification (VI-ReID) task. VI-ReID involves person retrieval across disjoint spectral cameras [10,11,12]. Given a visible (infrared) query image, the system matches a person of the same identity from the infrared (visible) gallery image. The 24/7 operational capability makes VI-ReID more suitable for practical applications. However, VI-ReID faces two key challenges: (1) modality differences caused by the imaging principles of visible and infrared images, and (2) intra-modality variations such as viewpoint, occlusion, pose, and illumination.

**Figure 1:**
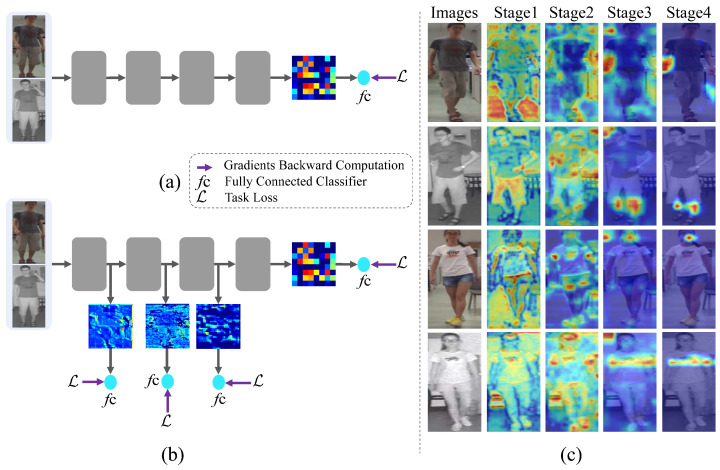
(**a**) The traditional supervised learning paradigm only imposes supervision on the last layer of a neural network. (**b**) Deep supervised learning involves training the last and intermediate layers concurrently. (**c**) Grad-CAM [21] visualization of attention maps at different feature extraction stages. Deeper red colors signify higher weights.

To address the above challenges. Previous works have approached these issues from multiple perspectives. Refs. [13,14,15,16] focus on designing two-stream networks that map modality-specific features to shared embedding subspaces to mitigate modality differences. Ref. [17] converted infrared images into visible images using a coloring approach, aiming to transform the cross-modality task into a single-modality problem to reduce the modality gap. Ref. [18] explored the use of GANs to generate intermediate modality images, compensating for features to reduce the gap between visible and infrared images. Recently, ref. [19] used semantic similarity to segment objects of the same category but with different appearances and backgrounds, proposing co-segmentation in multi-task learning to enhance VI-ReID. Ref. [20] introduced an enhanced contrastive loss to improve the learning of feature representations with high discriminability. However, as shown in Figure 1a, these studies primarily focus on learning shared feature representations in the final layer’s embedding space, neglecting the potential benefits of intermediate features. Intuitively, high-level semantic information at coarse resolution can limit feature representation, and the VI-ReID task becomes more challenging when color information is lost. As shown in Figure 1c, different stages emphasize distinct semantic information. In Stages 1 and 2, the model focuses on the body contour, while in Stages 3 and 4, it emphasizes high-level semantics such as the head and clothing patterns. Thus, low-level details can significantly enhance discriminative feature representation.

Based on the above analysis, refs. [22,23] explored utilizing intermediate layer features of deep supervised networks [24] to enhance the learning of modality-shared (or modality-invariant) features. Simultaneously, they mitigate modality differences and intra-modality variations by optimizing the intermediate layers, as shown in Figure 1b. However, these methods failed to achieve significant performance improvements due to the neglect of two key issues: (1) applying weak representation supervision loss to intermediate layers in advanced CNNs degrades task performance, and (2) multi-scale features lack sufficient information interaction.

**Figure 2:**
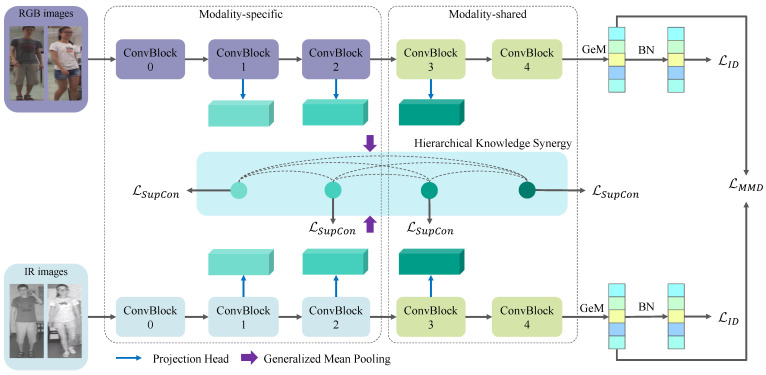
Illustration of the proposed MCLNet framework. MCLNet first decouples input IR and RGB images into modality-specific and modality-shared features. It then applies a generalized mean pooling (GeM) layer to generate a feature vector, followed by a batch normalization (BN) layer for identity inference. Meanwhile, the projection head maps multi-scale low-level features to the embedding space, where circles represent logits from the final layer and intermediate layers. Here, supervised contrastive learning (SCL) jointly supervises high-level semantics and low-level details while introducing a hierarchical knowledge synergy (HKS) strategy, using pairwise knowledge matching to enhance information consistency across supervised branches.

To address the above issues, we propose a multi-scale contrastive learning network (MCLNet) with hierarchical knowledge synergy for VI-ReID. MCLNet is a novel training framework with two-stream contrastive deep supervision for jointly training low-level details and high-level semantic information, as shown in Figure 2. Specifically, MCLNet first decouples input IR and RGB images into modality-specific and modality-shared features, extracting high-level semantic information in the modality-shared embedding space. Then, multi-scale low-level features are projected into the embedding space through a projection head. To capture multi-scale fine-grained features and fully utilize label information for improving the network’s comprehensive discriminative representation, we propose using supervised contrastive learning (SCL) for joint supervision of both high-level semantics and low-level details. Furthermore, we introduce a hierarchical knowledge synergy (HKS) strategy to facilitate explicit information interaction among all intermediate layers, thereby enhancing information consistency. HKS involves pairwise knowledge matching across all intermediate layers. We utilize modified KL-Divergence for pairwise matching of supervised branches to constrain their distribution. We extensively evaluated the effectiveness and generalization of MCLNet on three challenging benchmarks, SYSU-MM01 [9], RegDB [25], and LLCM [26].

The main contributions of this work are summarized as follows:

- We propose a multi-scale contrastive learning network (MCLNet) with hierarchical knowledge synergy for the VI-ReID task. This framework integrates supervision of low-level detail features and high-level semantic information to improve modality-invariant representation learning.
- We introduce supervised contrastive learning (SCL) for joint supervised training across intermediate layers, enhancing the network’s ability to learn superior visual representations. This approach reduces the feature distribution gap between modalities while increasing the separation between different classes.
- We utilize a hierarchical knowledge synergy (HKS) strategy for pairwise knowledge matching among intermediate layers, which further enhances the performance of VI-ReID.
- Extensive experiments on three publicly available datasets demonstrate the superiority of MCLNet compared to state-of-the-art methods.

The rest of this paper is organized as follows: Section 2 provides a comprehensive review of related work, followed by analysis and summarization. Section 3 introduces the proposed multi-scale contrastive learning network (MCLNet) with hierarchical knowledge synergy. Comprehensive performance evaluation is in Section 4. Finally, Section 5 offers a summary of the work and introduces future studies.

## 2. Related Work

### 2.1. Visible-Infrared Person Re-Identification

The modality difference between visible and infrared images presents a significant challenge for the VI-ReID task [9]. To address this issue, existing research primarily focuses on CNN-based methods [14,27,28,29,30,31,32,33,34,35,36], transformer-based methods [37,38,39,40] and generation techniques [12,41,42,43]. The VI-ReID task was first defined by [9], who also introduced deep zero-padding to convert visible and infrared images into two-channel images, thereby reducing modality differences. Refs. [27,28] developed a two-stream network to project modality-specific features into a shared feature space, learning robust and discriminative representations by embedding features. Their research significantly influenced subsequent work. Refs. [41,43] improved modality-invariant representation learning by generating cross-modality paired images, significantly bridging the modality gap. Ref. [30] explored the optimal number of shared parameters in the two-stream network architecture and proposed a hetero-center triplet loss to relax the strict constraints of traditional triplet loss, significantly enhancing VI-ReID performance. Ref. [35] developed a dual-attention mechanism within a two-stream network to align two modality-specific embedding spaces and facilitate local feature interaction, reducing cross-modality differences. Refs. [38,39] design feature extractors based on pure vision transformers to model long-range dependencies, aiming to capture comprehensive discriminative representations of person images. However, these methods only supervise the final layer of the network, overlooking the low-level details in intermediate layers that could enhance feature representation. In this work, we propose a multi-scale representation learning strategy that incorporates low-level features and high-level semantic information for joint supervised training.

### 2.2. Deep Supervision

As deep neural networks (DNNs) grow in depth and complexity, improvements in task performance are often accompanied by risks such as slow convergence and vanishing gradients. To address these issues, ref. [24] proposed deep supervised learning (DSL), which incorporates auxiliary supervision at each intermediate layer of the DNN. This approach seeks to accelerate network convergence and resolve the issue of vanishing gradients. Similarly, ref. [44] added two auxiliary classifiers to GoogLeNet to enhance the training process. Ref. [45] discussed using gradient-based heuristics to determine the optimal placement of auxiliary supervision. Additionally, refs. [46,47] proposed knowledge matching among all supervision branches to facilitate explicit information interaction. Recently, DSL has been applied to various visual tasks, such as medical image analysis [48,49], semantic segmentation [50], target detection [51], feature distillation [52], and single-modality person re-identification [53,54], achieving outstanding results.

In the VI-ReID task, prior studies [22,23,55] investigated using intermediate layer features of DSL to enhance modality-invariant feature learning, thereby reducing modality differences. However, these methods either merely fuse intermediate layer features or use a loss function with limited representation capability to supervise these layers, leading to constrained performance improvements. In this work, we propose a hierarchical knowledge synergy strategy to enhance information interaction between auxiliary branches and employ a more capable loss function to supervise intermediate layers. Experimental results confirm the effectiveness of our method and show promising performance improvements.

### 2.3. Contrastive Learning

In recent years, contrastive learning’s strong representation learning capability has made it increasingly popular in computer vision tasks [56,57,58,59,60]. Refs. [61,62] used instance discrimination as a contrastive learning paradigm for agent tasks, spurring further research on contrastive learning. Ref. [63] proposed the MoCo architecture, framing contrastive learning as a dictionary query problem and introducing queue and momentum encoders to enhance its effectiveness. To understand what drives contrastive prediction tasks to learn useful representations, ref. [64] proposed the SimCLP framework, highlighting the importance of large batch sizes and extended training durations in contrastive learning. SimSiam [65] investigated why strong performance can be achieved without negative samples, large batches, or momentum encoders. Currently, some studies [66,67] also apply contrastive learning to unsupervised VI-ReID tasks, exploring the potential of self-supervised learning in VI-ReID. Additionally, contrastive learning has demonstrated substantial efficacy in supervised learning. Ref. [68] introduced a supervised contrastive learning approach that utilizes label information to enhance contrastive learning’s representation capability. In this work, we propose a multi-scale contrastive learning network (MCLNet) grounded in deep supervised learning. While [69] showed that adding basic supervised losses to the intermediate layers of advanced CNNs may reduce performance. However, ref. [46] confirmed that carefully designed auxiliary supervision losses aid in regularizing modern CNNs and slightly improve accuracy. Therefore, we utilize supervised contrastive learning to guide the intermediate layers of MCLNet.

## 3. Methodology

### 3.1. Problem Formulation and Overview

Take D={V,R} to denote the set of cross-modality person images. $V={\left\{\left(x_{i}^{v},y_{i}^{v}\right)\right\}}_{i=1}^{N_{v}},R={\left\{\left(x_{i}^{r},y_{i}^{r}\right)\right\}}_{i=1}^{N_{r}}$ indicates the visible and infrared subsets with $N_{v}$ and $N_{r}$ samples, respectively. $x_{i}^{v}$ is the *i*-th visible sample and $y_{i}^{v}$ is the corresponding ground truth label. $x_{i}^{r}$ is the *i*-th infrared sample and $y_{i}^{r}$ is the corresponding ground-truth label. VI-ReID aims to match a given query visible (infrared) person image from the infrared (visible) gallery set. Existing works focus on learning task-relevant shared feature representations from the last layer of deep neural networks to mitigate the modality difference between visible and infrared images. However, high-level semantic features with coarse resolution and infrared features with missing color information weaken shared representation. Intuitively, low-level details can be a good complement to feature representation. Deep supervised learning is an effective approach for the VI-ReID task as it utilizes low-level details in the intermediate layer to enrich features while preserving high-level semantic information.

Figure 2 illustrates the architecture of the multi-scale contrastive learning network (MCLNet) with hierarchical knowledge synergy. MCLNet adopts a two-stream network as the feature extractor. Our backbone is ResNet-50 [70], which contains five convolution stages, indicating $\left\{S_{0},S_{1},S_{2},S_{3},S_{4}\right\}$. We follow [13] and set $\left\{S_{0},S_{1},S_{2}\right\}$ as modality-specific to extract specific features of the two modalities and $\left\{S_{3},S_{4}\right\}$ as modality-shared to extract discriminative shared features. $f^{v}$ and $f^{r}$ are feature representations extracted from visible and infrared images by modality-specific. MCLNet constructs a shared feature representation $f^{s}$ by constraining the diversity of feature distributions between the two modalities. We added auxiliary branches to each intermediate layer of MCLNet and implemented the same supervision to ensure consistent optimization objectives as the supervision of the entire framework.

### 3.2. Multi-Scale Representation

To take advantage of low-level details and high-level semantic information, we utilize a multi-scale representation strategy in deep supervised learning [24]. Specifically, we follow the symbol settings from the above subsection. Let $W_{c}$ be the weight of *L*-layer MCLNet that needs to be learned. When the supervised loss is exclusively applied to the final layer of the network, the optimization objective can be defined as:

(1) $$\underset{W_{c}}{\arg\min}L_{B}\left(W_{c},D\right)$$

where $L_{B}$ is the default loss of baseline [13], $L_{B}$ is defined as:

(2) $$L_{B}=L_{ID}\left(W_{c},D\right)+\lambda_{MMD}L_{MMD}\left(W_{c},D\right)$$

where $L_{ID}$ is a cross-entropy, $\lambda_{MMD}$ is a trade-off coefficient, following [13], $\lambda_{MMD}=0.25$. Maximum mean discrepancy (MMD) measures the closeness between two feature distributions [71]. However, directly applying the MMD distance can easily lead to overfitting and feature degradation [13]. Margin-based MMD $L_{MMD}$ is a variant of MMD that has the following advantages: (1) Only uses global features and does not rely on part-level features. (2) Align two modalities and class distributions at the same time. (3) Alleviates the overfitting problem. $L_{B}$ utilized a standard paradigm, the details of which we will not delve into in this paper.

In the VI-ReID task, the most currently popular methods employ the solution of implementing supervision at the last layer of the network. In contrast, our MCLNet employs deep supervised learning to add auxiliary supervision to all intermediate layers of the network for enhanced optimization during the training process. Let $W_{MR}=\left\{w_{MR}^{l}|l=1,2,\cdots ,L-1\right\}$ be the weight matrix that each intermediate layer of the network needs to learn, and $w_{MR}^{l}$ as the weight matrix of the *l*-th auxiliary supervision. In this work, $w_{MR}^{l}$ can be expressed as:

(3) $$w_{MR}^{l}=\left\{\begin{array}{cc} \left(w_{\mathrm{MR-v}}^{l},w_{\mathrm{MR-r}}^{l}\right)|l=1,2, & \mathrm{modality-specific}\\ w_{\mathrm{MR-s}}^{l}|l=3,4, & \mathrm{modality-shared} \end{array}\right.$$

where $w_{\mathrm{MR-v}}^{l},w_{\mathrm{MR-r}}^{l}$ indicates the weight matrix that needs to be learned for the visible and infrared auxiliary supervision, respectively. $w_{\mathrm{MR-s}}^{l}$ indicates the weight matrix that needs to be learned for auxiliary supervision in modality-shared. The optimization objective of the multi-scale representation learning strategy is the weighted sum of all auxiliary supervision losses and the supervision loss of the last layer, which can be expressed as:

(4) $$\underset{W_{c},W_{MR}}{\arg\min}L_{B}\left(W_{c},D\right)+\lambda_{MR}L_{MR}\left(W_{c},W_{MR},D\right)$$

where $\lambda_{MR}$ is the trade-off parameter, $L_{MR}$ is the weighted loss of multi-scale auxiliary supervision. $L_{MR}$ comprises two carefully designed loss functions applied simultaneously to the intermediate layers. The subsequent two subsections will offer a detailed explanation of these optimization objectives.

### 3.3. Supervised Contrastive Learning

Our method aims to exploit low-level details in intermediate layers to improve the learning of modality-invariant features, thereby alleviating modality differences. However, deep supervision forces the intermediate layer to learn task-related knowledge, which conflicts with the neural network’s feature extraction process. This paradox occasionally results in a reduction in the precision of the final task [72]. Therefore, it is necessary to employ a powerful loss function to supervise the intermediate layer. Conventional methods (e.g., cross-entropy) may not be the best choice for optimizing the intermediate layers. Meanwhile, to fully utilize the annotated information of the dataset, in this study, inspired by [68], we utilize supervised contrastive learning (SCL) to optimize the intermediate layer. The SCL learns different similarity representations from the same class while amplifying the differences between different classes. This characteristic enables the intermediate layer to learn better visual representations.

In a mini-batch of *N* sample pairs $\left\{\left(x_{1}^{v},x_{1}^{r}\right),\left(x_{2}^{v},x_{2}^{r}\right),\cdots ,\left(x_{n}^{v},x_{n}^{r}\right)\right\}$ consisting of visible and infrared images, we regard images with the same label as positive and images with different labels as negative. Let i∈I≡{1,⋯,N} be the index of any pairs. *z* denotes the normalized projection head output, which can be expressed as:

(5) $$z=\left\{\begin{array}{cc} \left(Proj\circ f_{i}^{v}\right)\|\left(Proj\circ f_{i}^{r}\right), & \mathrm{modality-specific}\\ Proj\circ f_{i}^{s}\circ \left(f_{i}^{v},f_{i}^{r}\right), & \mathrm{modality-shared} \end{array}\right.$$

where ∘ is the function composition operator, ‖ is element-wise concatenation, and Proj denotes the projection head. During training, each intermediate layer projection head maps the backbone features into the normalized embedding space. Given that the input features of the projection head are derived from intermediate layers rather than the final layer, it is crucial to project them correctly [73]. In this study, we enhance the complexity of the projection heads by using convolutional layers before nonlinear projections, as opposed to utilizing nonlinear projections stacked with fully connected layers and ReLU. The supervised contrastive learning loss can be expressed as:

(6) $$L_{SupCon}=\sum_{i\in I}\frac{-1}{\left|\begin{array}{c} P\left(i\right) \end{array}\right|}\sum_{p\in P\left(i\right)}\log\frac{\exp\left(z_{i}\cdot z_{p}/\tau \right)}{\sum_{a\in A\left(i\right)}\exp\left(z_{i}\cdot z_{a}/\tau \right)}$$

where τ is the temperature factor, following [68], τ=0.1. · denotes the inner (dot) product. P(i) is the set of all positives in the batch that are different from *i*, and $\left|\begin{array}{c} P(i) \end{array}\right|$ is its cardinality. A(i) is the set of samples in the batch except *i*. The loss function for the *l*-th layer is denoted as $L_{SupCon}\left(w_{MR}^{l},D\right)$, then our multi-scale representation supervised contrastive learning loss can be expressed as:

(7) $$L_{\mathrm{MR-SC}}\left(W_{MR},D\right)=\sum_{l=1}^{L-1}L_{SupCon}\left(w_{MR}^{l},D\right)$$

Our ablation study in Section 4 demonstrates that implementing supervised contrastive learning in the last layer of the network further improves performance. Therefore, we rewrite Equation (7) as:

(8) $$L_{\mathrm{MR-SC}}\left(W_{c},W_{MR},D\right)=\sum_{l=1}^{L}L_{SupCon}\left(W_{c},w_{MR}^{l},D\right)$$

### 3.4. Hierarchical Knowledge Synergy

In this subsection, we introduce the hierarchical knowledge synergy (HKS) strategy to enhance the interaction of explicit information across multi-scale features. HKS further advances the deep supervision learning paradigm from a novel perspective. Specifically, HKS utilizes embeddings generated by all normalized projection heads to guide network training. Its core contribution is a novel collaborative optimization strategy that facilitates dense pairwise bidirectional knowledge matching across all auxiliary branches, improving optimization efficiency. In this work, let $z_{m}$ and $z_{n}$ denote the normalized projection head output of the *m* and *n* branches, respectively. The pairwise knowledge matching between any two branches takes advantage of modified KL-Divergence to constrain their logical distribution, which can be expressed as:

(9) $$L_{\mathrm{MR-HKS}}\left(W_{c},W_{MR},D\right)=-\frac{1}{N}\sum_{i=1}^{N}\sum_{m=1}^{L}\sum_{n=1}^{L}\mu_{mn}z_{m}\log\frac{z_{m}}{z_{n}}$$

where $\mu_{mn}$ weights the pairwise knowledge matching of *m* and *n*, following [48], setting $\mu_{mn}=1$. HKS enables efficient mutual information interaction between the *m* and *n* branches, ensuring the dissemination of valuable knowledge across all auxiliary branches.

### 3.5. Overall Objective

Combining Equations (4), (8), and (9), the optimization objective of our MCLNet can be expressed as follows:

(10) $$\underset{W_{c},W_{MR}}{\arg\min}L_{B}\left(W_{c},D\right)+\lambda_{MR}\left(L_{\mathrm{MR-SC}}\left(W_{c},W_{MR},D\right)+L_{\mathrm{MR-HKS}}\left(W_{c},W_{MR},D\right)\right)$$

**Figure 3:**
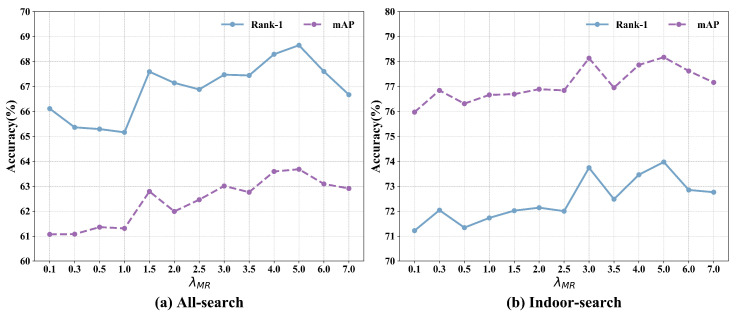
Analysis of trade-off coefficient $\lambda_{MR}$. Re-identification rates at Rank-1 (%) and mAP (%).

The ablation experiment on the setting of $\lambda_{MR}$ is shown in Figure 3 and the best performance was achieved when $\lambda_{MR}=5.0$.

## 4. Experiments

### 4.1. Datasets and Settings

#### 4.1.1. Datasets

The effectiveness of our method is evaluated on two publicly available real-world benchmarks, SYSU-MM01 [9], RegDB [25], and LLCM [26], respectively. The SYSU-MM01 dataset was captured by four visible and two infrared cameras in indoor and outdoor environments. The training set contains 22,258 RGB images and 11,909 IR images of 395 identities. The test set contains 3803 IR images of 96 identities for the query. The gallery has All-search or Indoor-search and single-shot according to the evaluation mode versions, details of each mode can be found in [14,34]. In this work, we adopt the All-search and Indoor-search evaluation modes in the most challenging and popularly utilized single-shot setting. The RegDB dataset contains 8240 images of 412 identities captured by a visible and an infrared camera, 206 identities for training, and the rest for testing. Each identity contains 10 RGB and 10 IR images. We alternately utilize RGB (IR) images for query (gallery) to evaluate the Visible-to-Infrared and Infrared-to-Visible modes. LLCM is a cross-modality dataset of 46,767 images from 1064 identities, captured using 9 cameras under challenging low-light conditions. It includes person images captured in various real-world scenarios, covering different climate conditions, low-resolution, and clothing styles. The training set includes 16,946 visible images and 13,975 near-infrared images from 713 identities, with the remaining 351 identities designated for testing. We alternately utilize visible (near-infrared) images for query (gallery) to evaluate the Visible-to-Infrared and Infrared-to-Visible search modes.

#### 4.1.2. Evaluation Protocols

Following standard protocols [9], query and gallery images come from different modalities. Standard cumulative matching characteristics (CMC) curves and mean average precision (mAP) are used for performance evaluation. We perform ten gallery set selection experiments and report the average performance.

#### 4.1.3. Implementation Details

The proposed method is implemented in PyTorch and trained on an NVIDIA GPU. ResNet-50 pre-trained on ImageNet [74] is used as the backbone network, and the stride of the last convolution block is set to 1 to obtain fine-grained feature maps [13]. All images were resized to 288 × 144, and the training set was random cropping, horizontal flipping, and random erasure (erasure probability ρ=0.5) for data augmentation. In the training phase, we adopt the warm-up strategy [34] and optimize using SGD with a momentum of 0.9 for a total of 80 epochs. The initial learning rate is set to 0.1 and decays by 0.1 and 0.01 at the 20 and 50 epochs, respectively. For sampling, we randomly sampled 4 identities, with 4 RGB and 4 IR images per person, for a total of 32 images per training batch. The code is available at https://github.com/qyhsxdx/MCLNet (accessed on 29 December 2024).

### 4.2. Comparison with State-of-the-Art Methods

In this subsection, we compare the proposed MCLNet with some state-of-the-art VI-ReID methods released in recent years, including CNN-based methods (DDAG [14], NFS [55], DMiR [10], G^2^DA [16], MTMFE [15], CSVI [19], FAM [35], DTRM [33], AGW [34], HC-Tri [30], CIA [20], LbA [75], CAJ [76]), generative-based methods (GECNet [17], JSIA-ReID [41], PAPG [43], TSME [12]), and transformer-based methods (PMT [38], TVTR [39], CMTR [40], SPOT [37]). Since these methods all follow standard evaluation protocols on three experimental datasets, we directly apply experimental results from published papers for comparison.

Table 1 compares our method with others on the SYSU-MM01 dataset, where MCLNet shows competitive results across all performance metrics. Compared with CNN-based methods, our approach achieves significant performance improvements. Specifically, compared with the suboptimal results of CIA methods, achieving Rank-1 accuracy improvements of 0.46% and 0.69% in two search modes, respectively. The comparative results indicate that our method’s ability to capture multi-scale features effectively enhances comprehensive discriminative representations. Compared to PAPG, which generates cross-modality paired images, our method achieves a 4.99% improvement in Rank-1 and a 2.69% gain in mAP under the All-search mode. In the Indoor-search mode, Rank-1 increases by 7.71%, while mAP improves by 6.62%. Especially, compared to the recent transformer-based method PMT, our approach performs worse in terms of mAP in the All-search mode. Attributable to the vision transformer’s capacity to model long-range dependencies, thereby improving global feature representations. However, MCLNet improved Rank-1 by 1.12%. In the Indoor-search mode, it achieved gains of 2.31% in Rank-1 and 1.65% in mAP. In conclusion, these results demonstrate the effectiveness of our method in the VI-ReID task.

The comparison results on the RegDB dataset are presented in Table 2. Due to better instance alignment and fewer intra-class variations in RegDB images [55], all methods show higher performance compared to SYSU-MM01. Our method achieves state-of-the-art performance in both search modes. Specifically, in the Visible-to-Infrared mode, Rank-1 and mAP show gains of 2.71% and 4.97%, respectively, compared to the suboptimal CSVI. In the Infrared-to-Visible mode, Rank-1 and mAP are improved by 3.65% and 5.73%, respectively. In addition, we observe that the performance gain of MCLNet compared with other methods is much larger than that obtained on SYSU-MM01. In conclusion, these results demonstrate that MCLNet exhibits superior robustness and generalization.

Moreover, to further validate the scalability of MCLNet, we performed comprehensive experiments on the large-scale, complex low-light LLCM dataset, as shown in Table 3. Our method achieves optimal results in both search modes. Compared to the strong baseline CAJ, our method achieves improvements of 0.43% in Rank-1 and 0.65% in mAP for the Visible-to-Infrared mode. In the Infrared-to-Visible mode, it demonstrates gains of 0.86% in Rank-1 and 0.09% in mAP. In conclusion, the experimental results confirm that MCLNet exhibits robust performance and strong generalization in complex real-world datasets.

### 4.3. Ablation Study

In this subsection, we examine the impact of supervision applied by MCLNet at different convolutional stages. Additionally, we evaluate the effectiveness of various MCLNet components across both search modes on SYSU-MM01 and RegDB. B indicates the baseline with the learning objective $L_{B}$. S and H indicate components with the learning objectives $L_{\mathrm{MR-SC}}$ and $L_{\mathrm{MR-HKS}}$, respectively.

#### 4.3.1. Impact of Supervision Scope

In this part, we compare the performance of different convolutional stages of MCLNet, where ${\left\{S_{i}\right\}}_{i=1}^{4}$ denotes the *i*-th convolutional stage, as shown in Table 4. Rank-1 and mAP in both search modes tend to improve when supervision is applied separately at each convolutional stage. The latter is superior to the former when we perform supervision in the modality-specific $\left\{S_{1},S_{2}\right\}$ and modality-shared $\left\{S_{3},S_{4}\right\}$, respectively. These findings indicate that our method effectively learns modality-invariant feature representations in the embedding space. Notably, the best results are achieved when supervision is simultaneously applied across all convolutional stages. This improvement may be attributed to HKS facilitating information interaction between low-level details and high-level semantics, enhancing information consistency. However, indiscriminately applying joint supervision to intermediate layers (e.g., rows 6 and 7) may pose significant challenges to visual representation and knowledge synergy.

#### 4.3.2. Evaluation of Different Loss Components

Table 5 shows the performance improvements achieved by each component on the baseline learning objective $L_{B}$. To ensure consistency, all hyperparameters were kept constant during evaluation. As shown in Table 5, B+S compared to B, supervised contrastive learning (SCL) achieved gains of 3.19% Rank-1 and 3.20% mAP in the All-search mode. Similar improvements were observed in both search modes on the RegDB dataset. These comparative results indicate the superiority of SCL in enhancing feature representation learning. Furthermore, we investigated the contribution of hierarchical knowledge synergy (HKS) is shown in the 3-th row of Table 5. Compared to B, B+H shows significant improvements of 1.42% in Rank-1 and 1.05% in mAP on the SYSU-MM01 dataset in the All-search mode. Notably, on the RegDB dataset, Rank-1 is slightly lower than the baseline in both search modes, while mAP shows improvements of 0.41% and 1.40%, respectively. The comparative results demonstrate that HKS improves multi-scale feature interaction, leading to enhanced retrieval performance in the network architecture.

We further validated the effectiveness of joint supervision by SCL and HKS, with the results presented in the 4-th row of Table 5. Performance improvements are evident in both search modes on SYSU-MM01, indicating that HKS enhances information consistency and helps MCLNet learn more discriminative features. Notably, on the RegDB dataset, B+S+H shows lower performance on most metrics compared to B+S, except for an increase in Rank-1 under the Visible-to-Infrared search mode. We attribute this to HKS acting as a powerful regularizer that suppresses noise interference [46], leading to overfitting on the simpler and more blurred contours of RegDB images captured by far-infrared cameras. Overall, the performance gains across different datasets confirm the effectiveness of our method.

### 4.4. Visualization Analysis

In this subsection, we studied the influence of the trade-off coefficient $\lambda_{MR}$ in the optimization objective Equation (10). We perform an analysis of the similarity of intra-person and inter-person features learned by MCLNet. Meanwhile, Grad-CAM visualization of attention maps of different components is performed. We also show the retrieval results of Rank-10. All experiments are implemented on SYSU-MM01.

#### 4.4.1. Influence of Hyperparameters

We evaluated the effect of the trade-off parameter $\lambda_{MR}$ on performance. Figure 3 shows the analysis of how different values of $\lambda_{MR}$ impact two key evaluation metrics (Rank-1, mAP) in both All-search and Indoor-search modes. Independent experiments were conducted by gradually increasing $\lambda_{MR}$ from 0.1 to 7.0. As shown in Figure 3, varying $\lambda_{MR}$ led to consistent improvements, with both metrics initially rising and then declining. The optimal Rank-1 and mAP accuracies were achieved with $\lambda_{MR}$ set at 5.0 in both search modes.

#### 4.4.2. Visualization of Similarity Distribution

**Figure 4:**
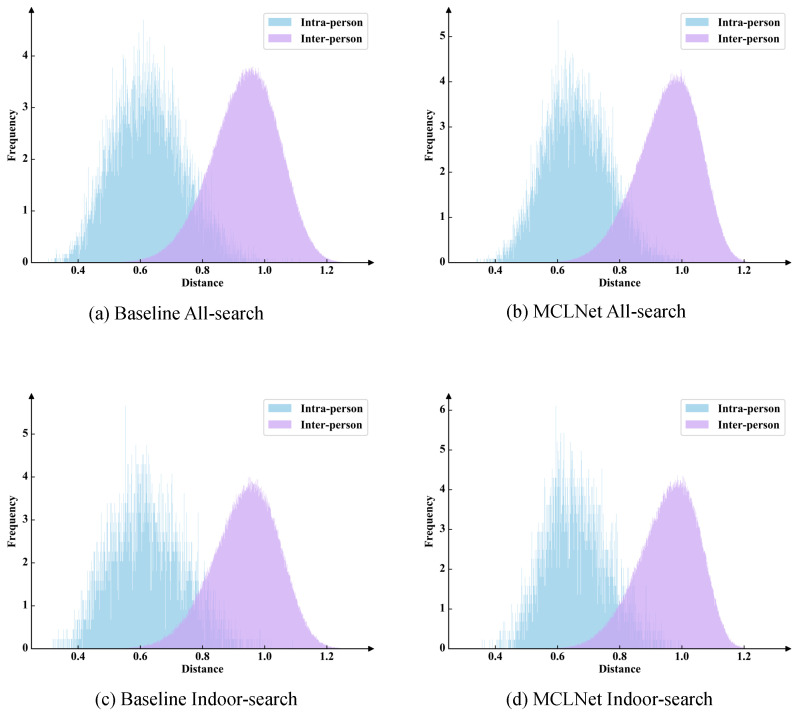
The distribution of intra-person and inter-person similarities on the two search modes of the SYSU-MM01 dataset.

To further verify the effectiveness of our multi-scale contrastive learning method with hierarchical knowledge synergy, we visualized the intra-person and inter-person similarity distributions for the baseline and MCLNet across two search modes, as shown in Figure 4. Similarities were measured using cosine distance. Specifically, Figure 4a,c illustrate the similarity distributions of features extracted by the baseline method in both search modes, while Figure 4b,d show the distributions for features extracted by MCLNet. Compared to Figure 4a, Figure 4b shows a significant reduction in the overlap of the similarity distribution, with intra-person similarities becoming more concentrated, while inter-person similarities remain unchanged. This trend is also observed in the Indoor-search mode. These results demonstrate that our method effectively bridges modality gaps and reduces intra-class variations in cross-modality settings.

#### 4.4.3. Visualization of Feature Heatmaps

**Figure 5:**
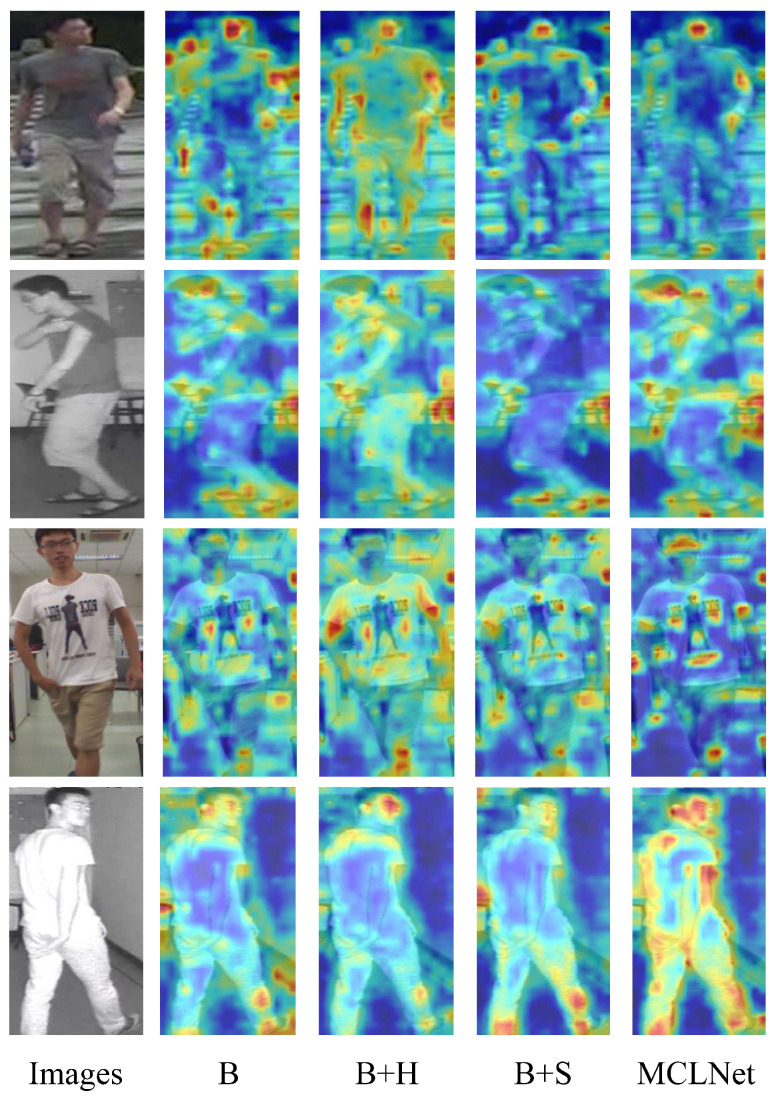
Visualization results of different modality images of randomly selected two identities. Grad-CAM [21] visualization of the attention maps of B, B + H, B + S, and MCLNet methods are performed, respectively. Deeper red colors signify higher weights.

We compare the attention maps of different components, as shown in Figure 5. In this figure, B indicates the baseline, while H and S denote hierarchical knowledge synergy and supervised contrastive learning, respectively. The analysis yields the following conclusions: (1) While the baseline method enhances various regions of the person, it also focuses excessively on background noise. In contrast, our method highlights identity-related regions and reduces attention to noisy areas. For instance, Figure 5’s last column shows that MCLNet effectively focuses on the subject’s non-background regions, even when the background is prominent. (2) Compared to B + S and B + H, MCLNet emphasizes more discriminative features. The last two rows of Figure 5 illustrate that MCLNet concentrates on clothing patterns and the presence of glasses for identity discrimination, while also paying increased attention to body parts. These results indicate that our method enhances modality-invariant feature representation, improves intra-class similarity, and effectively mitigates modality discrepancies in VI-ReID.

#### 4.4.4. Top Retrieved Examples

**Figure 6:**
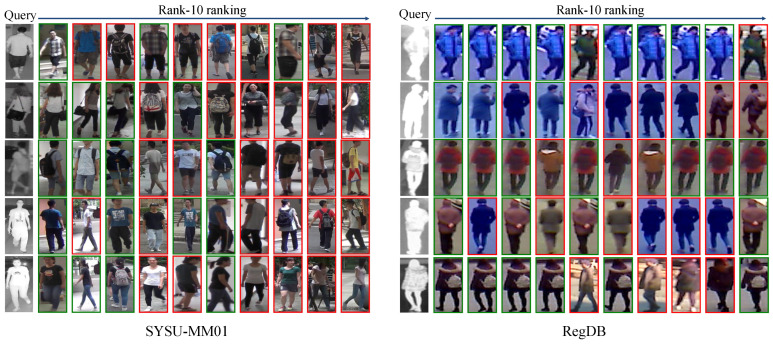
Top-10 retrieved results of some example queries with the MCLNet on SYSU-MM01 and RegDB. The green and red bounding boxes indicate query results matching the same identity and different identities from the gallery, respectively.

Figure 6 shows the top 10 retrieval results for five randomly selected query examples on the SYSU-MM01 and RegDB datasets. It is evident that even humans struggle to correctly match the same identity using only color and body shape information (e.g., Rank-5 and Rank-6 in the 4-th and 5-th rows on SYSU-MM01). This highlights the extreme challenge but also the importance of VI-ReID tasks, particularly in nighttime surveillance. Notably, our MCLNet demonstrates strong performance, accurately matching the same identity and different identities in the gallery across all five randomly selected queries. These visualization results confirm the effectiveness and superiority of our MCLNet.

### 4.5. Applicability to Other Methods

In this subsection, we apply the proposed multi-scale contrastive learning method with hierarchical knowledge synergy to the VI-ReID models AGW [34] and DDAG [14] to evaluate its generalization across different methods. We maintained the original parameter settings of AGW and DDAG and conducted experiments in both search modes on the SYSU-MM01 and RegDB datasets. Table 6 clearly demonstrates that our method significantly enhances the performance scores of various VI-ReID models. Specifically, when applied to AGW in the All-search mode of SYSU-MM01, our method achieved gains of 3.85% in Rank-1 and 2.12% in mAP. In the Indoor-search mode, when applied to DDAG, it achieved gains of 5.24% in Rank-1 and 3.64% in mAP. The performance improvement is particularly significant in the RegDB datasets. In the Visible-to-Infrared mode, when our method is applied to AGW, Rank-1 and mAP increase by 15.29% and 15.53%, respectively. These gains clearly illustrate the effectiveness of our method in enhancing shared feature representation and improving information consistency. Additionally, they demonstrate MCLNet’s strong generalization and robustness in the VI-ReID challenge.

## 5. Conclusions and Future Work

In this paper, we reassess the effectiveness of deep supervised learning in the VI-ReID task and propose a novel multi-scale contrastive learning network (MCLNet) with hierarchical knowledge synergy (HKS) for joint supervision of low-level details and high-level semantic information. MCLNet introduces supervised contrastive learning (SCL) to supervise intermediate layers, enhancing cross-modality feature representation. Additionally, we employ a hierarchical knowledge synergy (HKS) strategy, incorporating dense pairwise knowledge matching between supervised branches to enhance information consistency. Extensive experiments on three challenging benchmarks demonstrate the effectiveness and generalizability of our method.

The ablation study indicates that HKS may cause overfitting when training networks on images with simple scenes and blurred contours. We will investigate methods to mitigate HKS overfitting. In addition, comparative results highlight the superior capability of vision transformers in modeling long-range dependencies to improve VI-ReID retrieval performance. Consequently, future research will focus on hybrid CNN and transformers to effectively capture both local features and global context information.

## Figures and Tables

**Table 1:** Comparison with state-of-the-art methods on the SYSU-MM01 dataset in two search modes. Re-identification rates at Rank-1 (%) and mAP (%).

Method	Classification of Methods	All-Search		Indoor-Search	
		Rank-l	mAP	Rank-20	mAP
AGW [34]	CNN-based Methods	47.50	47.65	54.17	62.97
NFS [55]		56.91	55.45	62.79	69.79
DTRM [33]		63.03	58.63	66.35	71.76
DMiR [10]		50.54	49.29	53.92	62.49
CIA [20]		68.19	64.82	73.28	77.49
G^2^DA [16]		57.07	55.05	63.70	69.83
FAM [35]		55.75	51.52	58.24	65.65
DDAG [14]		54.75	53.02	61.02	67.98
HC-Tri [30]		61.68	57.51	63.41	68.17
MTMFE [15]		62.56	60.57	65.06	73.86
GECNet [17]	Generative-based Methods	53.37	51.83	60.60	62.89
JSIA-ReID [41]		38.10	36.90	43.80	52.90
TSME [12]		64.23	61.21	64.80	71.53
PAPG [43]		63.66	60.99	66.26	71.55
SPOT [37]	Transformer-based Methods	65.34	62.25	69.42	74.63
PMT [38]		67.53	64.98	71.66	76.52
TVTR [39]		65.30	64.15	72.21	77.94
CMTR [40]		65.45	62.90	71.46	76.67
MCLNet (Ours)	CNN-based Methods	68.65	63.68	73.97	78.17

**Table 2:** Comparison with state-of-the-art methods on the RegDB dataset in two search modes. Re-identification rates at Rank-1 (%) and mAP (%).

Method	Classification of Methods	Visible-to-Infrared		Infrared-to-Visible	
		Rank-l	mAP	Rank-l	mAP
AGW [34]	CNN-based Methods	70.05	66.37	70.49	65.90
NFS [55]		80.54	72.10	77.95	69.79
DTRM [33]		79.09	70.09	78.02	69.56
DMiR [10]		75.79	69.97	73.93	68.22
CIA [20]		89.73	82.78	87.71	80.79
G^2^DA [16]		71.72	65.90	69.50	63.88
FAM [35]		87.31	76.70	84.81	74.73
DDAG [14]		69.34	63.46	68.06	61.80
HC-Tri [30]		91.05	83.29	89.30	81.46
MTMFE [15]		76.10	74.39	72.18	71.04
CSVI [19]		91.41	85.14	90.06	83.86
GECNet [17]	Generative-based Methods	82.33	78.45	78.93	75.58
JSIA-ReID [41]		48.50	49.30	48.10	48.90
TSME [12]		87.35	76.94	86.41	75.70
PAPG [43]		88.35	83.18	86.46	80.08
SPOT [37]	Transformer-based Methods	80.35	72.46	79.37	72.26
PMT [38]		84.83	76.55	84.16	75.13
TVTR [39]		84.10	79.50	83.70	78.00
CMTR [40]		88.11	81.66	84.92	80.79
MCLNet (Ours)	CNN-based Methods	94.12	90.11	93.71	89.59

**Table 3:** Comparison with state-of-the-art methods on the LLCM dataset in two search modes. Re-identification rates at Rank-1 (%) and mAP (%).

Method	Classification of Methods	Visible-to-Infrared		Infrared-to-Visible	
		Rank-l	mAP	Rank-l	mAP
DDAG [14]	CNN-based Methods	48.50	53.00	41.00	49.60
AGW [34]		56.00	59.10	46.40	54.80
LbA [75]		50.80	55.90	44.60	53.80
CAJ [76]		56.50	59.80	48.80	56.60
MCLNet (Ours)	CNN-based Methods	56.93	60.45	49.66	56.69

**Table 4:** Comparison of supervision implemented at different convolutional stages in MCLNet. Re-identification rates at Rank-1 (%) and mAP (%).

Method	$S_{1}$	$S_{2}$	$S_{3}$	$S_{4}$	All-Search		Indoor-Search	
					Rank-l	mAP	Rank-l	mAP
1	✓				65.19	60.28	69.30	74.33
2		✓			66.46	61.43	72.46	76.78
3			✓		68.08	63.02	73.45	77.42
4				✓	68.01	63.19	72.42	77.43
5	✓	✓			66.60	61.87	73.54	77.54
6	✓	✓	✓		65.70	61.30	71.73	76.31
7	✓	✓		✓	65.62	60.82	70.91	75.70
8	✓	✓	✓	✓	68.65	63.68	73.97	78.17
9			✓	✓	68.09	63.05	73.31	77.64

**Table 5:** Evaluation of different components in two search modes on SYSU-MM01 and RegDB. Re-identification rates at Rank-1 (%) and mAP (%).

Method	SYSU-MM01				RegDB			
	All-Search		Indoor-Search		Visible-to-Infrared		Infrared-to-Visible	
	Rank-1	mAP	Rank-1	mAP	Rank-1	mAP	Rank-1	mAP
B	64.86	60.12	-	-	93.95	88.74	93.57	86.54
B+S	68.05	63.32	73.04	77.41	94.12	90.11	93.71	89.59
B+H	66.28	61.17	71.78	76.18	93.88	89.15	92.44	87.94
B+S+H	68.65	63.68	73.97	78.17	94.38	90.02	92.55	86.83

**Table 6:** Our method evaluates AGW and DDAG in two search modes on SYSU-MM01 and RegDB. Re-identification rates at Rank-1 (%) and mAP (%).

Method	SYSU-MM01				RegDB			
	All-Search		Indoor-Search		Visible-to-Infrared		Infrared-to-Visible	
	Rank-1	mAP	Rank-1	mAP	Rank-1	mAP	Rank-1	mAP
AGW [34]	47.50	47.65	54.17	62.97	70.05	66.37	70.49	65.90
+MCLNet	51.35	49.77	57.16	64.76	85.34	81.90	83.65	80.70
(Ours)	↑3.85	↑2.12	↑2.99	↑1.79	↑15.29	↑15.53	↑13.16	↑14.80
DDAG [14]	54.75	53.02	61.02	67.98	69.34	63.46	68.06	61.80
+MCLNet	58.35	54.33	66.26	71.62	80.43	76.26	76.28	72.43
(Ours)	↑3.60	↑1.31	↑5.24	↑3.64	↑11.09	↑12.80	↑8.22	↑10.63

## Data Availability

Data will be made available on request.
